# Automated Extraction of Diagnostic Criteria From Electronic Health Records for Autism Spectrum Disorders: Development, Evaluation, and Application

**DOI:** 10.2196/10497

**Published:** 2018-11-07

**Authors:** Gondy Leroy, Yang Gu, Sydney Pettygrove, Maureen K Galindo, Ananyaa Arora, Margaret Kurzius-Spencer

**Affiliations:** 1 University of Arizona Tucson, AZ United States

**Keywords:** parser, natural language processing, complex entity extraction, Autism Spectrum Disorder, DSM, electronic health records, decision tree, machine learning

## Abstract

**Background:**

Electronic health records (EHRs) bring many opportunities for information utilization. One such use is the surveillance conducted by the Centers for Disease Control and Prevention to track cases of autism spectrum disorder (ASD). This process currently comprises manual collection and review of EHRs of 4- and 8-year old children in 11 US states for the presence of ASD criteria. The work is time-consuming and expensive.

**Objective:**

Our objective was to automatically extract from EHRs the description of behaviors noted by the clinicians in evidence of the diagnostic criteria in the Diagnostic and Statistical Manual of Mental Disorders (DSM). Previously, we reported on the classification of entire EHRs as ASD or not. In this work, we focus on the extraction of individual expressions of the different ASD criteria in the text. We intend to facilitate large-scale surveillance efforts for ASD and support analysis of changes over time as well as enable integration with other relevant data.

**Methods:**

We developed a natural language processing (NLP) parser to extract expressions of 12 DSM criteria using 104 patterns and 92 lexicons (1787 terms). The parser is rule-based to enable precise extraction of the entities from the text. The entities themselves are encompassed in the EHRs as very diverse expressions of the diagnostic criteria written by different people at different times (clinicians, speech pathologists, among others). Due to the sparsity of the data, a rule-based approach is best suited until larger datasets can be generated for machine learning algorithms.

**Results:**

We evaluated our rule-based parser and compared it with a machine learning baseline (decision tree). Using a test set of 6636 sentences (50 EHRs), we found that our parser achieved 76% precision, 43% recall (ie, sensitivity), and >99% specificity for criterion extraction. The performance was better for the rule-based approach than for the machine learning baseline (60% precision and 30% recall). For some individual criteria, precision was as high as 97% and recall 57%. Since precision was very high, we were assured that criteria were rarely assigned incorrectly, and our numbers presented a lower bound of their presence in EHRs. We then conducted a case study and parsed 4480 new EHRs covering 10 years of surveillance records from the Arizona Developmental Disabilities Surveillance Program. The social criteria (A1 criteria) showed the biggest change over the years. The communication criteria (A2 criteria) did not distinguish the ASD from the non-ASD records. Among behaviors and interests criteria (A3 criteria), 1 (A3b) was present with much greater frequency in the ASD than in the non-ASD EHRs.

**Conclusions:**

Our results demonstrate that NLP can support large-scale analysis useful for ASD surveillance and research. In the future, we intend to facilitate detailed analysis and integration of national datasets.

## Introduction

Based on data from autism spectrum disorder (ASD) surveillance, it is estimated that the prevalence of ASD is approximately 1.5% [1]. In the second half of the 20^th^ century, it was estimated at slightly more than 5 cases per 10,000 people. Since the 1990s, however, measured prevalence has increased [2]. In 2000, prevalence estimates ranged from 4.5 to 9.9 cases per 1000 children and increased to 1 in 110 children in 2006 [3] and 1 in 59 in 2014 [4]. The reasons for this trend are uncertain, but the following factors have been proposed: increased public awareness, changing diagnostic criteria, and substitution of ASD eligibility for other special education eligibilities as well as the possibility that the true prevalence of ASD is increasing [3,4].

Data on long-term trends, symptoms, diagnoses, evaluations, and treatments are critical for planning interventions and educational and health services. To understand and act upon such trends, large-scale studies are needed that can evaluate trends over time, integrate different types of data, and review large datasets. In recent years, data have been increasingly electronically encoded in electronic health records (EHRs) in structured fields and free text. Collection of such EHRs enables analyses that compare and contrast ASD prevalence in relation to other variables and over time.

Much of the published work on ASD leverages information in the structured fields of the EHRs such as gender, medication taken by the mother, birth complications, scores on a variety of tests, and others. The structured data portions are relatively easy to extract and are useful for large-scale studies. However, the results of the analysis are commonly limited to reviews and counts of the presence of conditions in certain populations [5]. For example, Clements et al [6] evaluated the relationship between autism and maternal use of prenatal antidepressants using EHRs.

EHR of people with ASD contain extensive free text fields with important information that is often complementary to and more detailed and explanatory than the structured data. This is because in the absence of any biological laboratory test, diagnosis is generally made in person using specific test instruments, history, and differential diagnosis, and much of this information is recorded as narrative. Automatically extracting this information from the EHRs requires natural language processing (NLP). So far, a few NLP approaches have been used to analyze language generated by people on the spectrum [7,8], but there has been little focus on the text in EHRs.

The existing projects that focus on the text in EHRs fall into two groups. The first group focuses on using all the free text combined with structured fields to automatically assign case status (classification of patients as cases of autism or not) to an entire record. A variety of machine learning algorithms are useful for this task. Using a subset of the EHRs for training, these algorithms create a model that can be applied to future EHRs to assign case status. These models can be human-interpretable, such as decision trees, or can be black box approaches, such as neural networks. In our own work [9], we compared decision trees (C5.0) and a feedforward backpropagation artificial neural network using only the information contained in the free text. Our best approach used the decision tree and was 87% accurate in case assignment. Similarly, promising results (86% accuracy) were achieved by Maenner et al [10] using a random forest algorithm. Lingren et al [11] used International Classification of Diseases (ICD)-9 codes combined with concepts extracted from the free text and compared rule-based and machine learning algorithms with similarly good results.

In addition to case status assignment, more detailed use of the information contained in the free text would be helpful for large-scale analysis, for example, cultural comparisons as suggested by Mandy et al [12], and for combination with other data. To extract this information automatically, a comprehensive set of tools is needed [13,14] for standard NLP tasks, such as part-of-speech (POS) tagging and grammatical parsing. For more specialized tasks, such as concept detection, entity and relation extraction, and coreference resolution, we work on entity extraction algorithms. In medicine, existing entity extraction algorithms focus on different types of text (eg, published research abstracts or clinical narrative) and can be rule-based or use machine learning techniques. The entities themselves have been predominantly single terms or relationships composed of single terms. For example, several projects focus on annotating diseases or genes and proteins [15-18] and the biomedical relationships between them [19,20]. When working with free text from EHRs, a variety of entities have been the focus. For example, NLP for safety surveillance by extracting information on postoperative complications [21] and adverse drug effects from psychiatric records [22], clinical event detection (eg, fever, change in output) for transcriptions of the handoff communication between nurses during shift changes [23,24], and even the creation of new data such as veterans’ employment information [25].

In this project, we aimed to extract the expressions of the behaviors indicative of individual diagnostic criteria as described in the Diagnostic and Statistical Manual of Mental Disorders, Fourth Edition, Text Revision (DSM-IV-TR) [26]. Such expressions are more complex than single terms, and each diagnostic criterion can be expressed by a variety of different behaviors described in a diverse manner in the text. We envision that our parser will be useful for two types of work. First, it will enable autism surveillance to speed up and increase the scope when processing school and health records. Currently, the Centers for Disease Control and Prevention (CDC) surveillance effort is limited to 11 states and a subset of schools, the catchment area, in these states. By automating the review of records, an efficient approach will allow all records to be reviewed, many case decisions will be automated, and only a subset of records will require review by experienced clinicians. A second important use that we foresee is in the creation of large sets of structured data from the information available in the EHRs. This will facilitate secondary analysis of data on its own and in combination with other data sources at a previously unseen scale.

We first describe the development and evaluation of the parser, including important decisions on using off-the-shelf tools and machine learning algorithms. Then, we present a case study where we applied our parser to almost 5000 EHRs to demonstrate usefulness for detailed analysis over time.

## Methods

### Overview

Our parser uses human-interpretable rules to match complex patterns that represent the DSM diagnostic criteria. These rule-based algorithms rely on the creation of patterns of terms, grammatical relationships, and the surrounding text to recognize the entities of interest in text.

### Records and the Diagnostic and Statistical Manual of Mental Disorders Criteria

We work with EHRs created by the Arizona Developmental Disabilities Surveillance Program (ADDSP) as part of the CDC multicenter Autism and Developmental Disability Monitoring Network surveillance. Our ADDSP records are collected from educational and clinical data sources in 11-15 school districts for 8-year olds. From 2000 to 2010, a total of 27,515 records were reviewed and 6176 records were abstracted that included any of the 32 social behavioral triggers consistent with ASD as listed in the Abstraction Manual developed by the CDC. These records referred to 4491 children. The identified records for each child were further evaluated by trained clinical reviewers who applied standardized criteria to highlight criteria and determine ASD case status. This yielded 2312 confirmed cases.

We have access to the records and the case status of each child as determined through expert review of the information. For this study, we leveraged a subset of these records (n=93) that have been printed and have the diagnostics criteria annotated on this paper version. The electronic version does not include markings indicating the criteria. Therefore, we first created an electronic gold standard with all information combined. Records were loaded using WebAnno [27], and the annotations made by clinicians on the paper versions were added to the electronic versions. In 1 hour, 1-3 records could be annotated depending on the length of the record.

We intend to automate the extraction of the DSM-IV-TR [26] criteria for ASD. Textbox 1 shows example criteria rules. The DSM specifies the combination of criteria needed to assign ASD case status. While other instruments exist for diagnosing autism, as well as different versions of the DSM, we focus on matching to the DSM-IV-TR because this is an approach that is used worldwide and that is sometimes used for matching to billing codes (ICD-9 and ICD-10). It is also available with a large set of records for training and testing. Later, we will work with the DSM-V, the newest version.

**Example rules in Diagnostic and Statistical Manual of Mental Disorders, Fourth Edition, Text Revision, to diagnose autistic disorder.**

Rules

A: A total of 6 or more items from (1), (2), and (3), with at least 2 from (1) and 1 each from (2) and (3):

- 1: Qualitative impairment in social interaction, as manifested by at least 2 of the following:
  - A1a: Marked impairment in the use of multiple nonverbal behaviors such as eye-to-eye gaze, facial expression, body postures, and gestures to regulate social interaction
  - A1b: Failure to develop peer relationships appropriate to developmental level
  - A1c: A lack of spontaneous seeking to share enjoyment, interests, or achievements with other people
  - A1d: Lack of social or emotional reciprocity

- 2: Qualitative impairments in communication as manifested by at least 1 of the following:
  - A2a: Delay in, or total lack of, the development of spoken language (not accompanied by an attempt to compensate through alternative modes of communication such as gesture or mime)
  - A2b: In individuals with adequate speech, marked impairment in the ability to initiate or sustain a conversation with others
  - A2c: Stereotyped and repetitive use of language or idiosyncratic language
  - A2d: Lack of varied, spontaneous make-believe play or social imitative play appropriate to developmental level

- 3: Restrictive, repetitive, and stereotyped patterns of behaviors, interests, and activities, as manifested by at least 1 of the following:
  - A3a: Encompassing preoccupation with 1 or more stereotyped and restricted patterns of interest that is abnormal either in intensity or focus
  - A3b: Apparently inflexible adherence to specific, nonfunctional routines or rituals
  - A3c: Stereotyped and repetitive motor mannerisms
  - A3d: Persistent preoccupation with parts of objects

### Design Choices

#### Component Selection Rationale

To our knowledge, no parsers exist that identify DSM criteria in EHRs. As part of our development, we evaluated MetaMap [28,29] for use as an off-the-shelf building block. MetaMap maps terms to the Unified Medical Language System (UMLS) Metathesaurus concepts and semantic types.

Using 2 EHRs from our development set, we analyzed MetaMap’s outcome in the context of ASD. From the 2 EHRs, a total of 259 phrases were extracted and mapped to 632 UMLS concepts. Overall, 46.5% (294/632) of all candidate mappings for those phrases were correct and useful for our domain; 55.2% (143/259) of phrases were given a single candidate mapping to UMLS concepts, and for those single matches, the accuracy was high, with 81.1% (116/143) correct and useful matches for our domain. However, when the number of matched semantic types increases, it becomes increasingly complicated to identify the correct concept and associated semantic type. Furthermore, the majority of semantic types do not apply to our domain. Using a very lenient approach, we consider approximately 31 semantic types useful to match to DSM criteria (eg, Activity, Anatomical Structure, Behavior, Body Part, Organ or Organ Component, Body Substance, Clinical Attribute, Conceptual Entity, and Daily or Recreational Activity, among others). Although the 259 phrases we analyzed are restricted to 31 relevant semantic types, this is not enough to distinguish ASD diagnostic criteria from rest of the text: only 27.0% (70/259) phrases intersect with ASD diagnostic criteria. Because the number of types that are immediately useful is small and this MetaMap outcome would require significant development to adjust for our purpose, building an extraction system on top of this is impractical. Therefore, we decided to build all the components in-house.

#### Rule-based Versus Machine Learning Rationale

When developing a new entity extraction artifact, a rule-based or machine learning approach is chosen as the starting point. Both can be combined in ensemble methods later. We performed a baseline test using a decision tree, which was chosen because it is a human-interpretable machine learning algorithm.

We formulated the problem as a multiclass sentence classification problem (12 diagnostic labels or null label). We used Stanford CoreNLP (version 3.7.0) for NLP processing. We used a standard bag-of-words approach with and trained the algorithm on 120 records containing 19,428 sentences. Because our records contain approximately 0.5%-5% sentences describing a DSM criterion, we undersampled negative examples during training to improve recall: for each positive example, we sampled 30 negative samples (except criteria A2a and A2b, which occurred frequently enough to use on the entire training data). Our features were lemmas, as determined by CoreNLP, which appeared more than 5 times in the training data (2913 terms). We used a pruned decision tree (Weka version 3.8.0) with a pruning confidence threshold of 0.25. Size of the vocabulary, undersampling ratio, and pruning threshold were determined based on the best values we found during exploration.

Table 1 shows the results for classification at the sentence level. For comparison, we applied the model to the same EHR test set (not used during decision tree training) used in our parser evaluation below. Overall, *F*-score was <0.5. Neither precision nor recall was high using this approach.

This machine learning approach will require significant work to improve performance. We believe this cannot be attained with simple changes in the input, such as word embedding, or by changing algorithms. It will require more sophisticated features and a much larger dataset. We, therefore, first created a rule-based parser, which may provide better results overall as well as insights related to lexicons and features useful for future combinations with machine learning in a classifier ensemble.

**Table 1:** Decision tree evaluation for sentence classification.

Rule	Count of positive cases	% positive cases (of all sentences)	Precision	Recall	F-score	Specificity
A1a	120	0.021	0.70	0.52	0.59	0.99
A1b	91	0.016	0.50	0.42	0.45	0.99
A1c	35	0.006	0.16	0.17	0.17	0.99
A1d	160	0.029	0.54	0.14	0.22	1.00
A2a	388	0.069	0.71	0.39	0.50	1.00
A2b	321	0.057	0.69	0.37	0.48	0.99
A2c	120	0.021	0.54	0.47	0.51	0.99
A2d	62	0.011	0.34	0.19	0.25	1.00
A3a	64	0.011	0.20	0.09	0.13	1.00
A3b	123	0.022	0.81	0.47	0.59	1.00
A3c	66	0.012	0.70	0.32	0.44	1.00
A3d	27	0.005	0.27	0.30	0.28	1.00
Microaverage	1577	0.024	0.60	0.35	0.45	0.99

**Table 2:** Lexicon overview.

Pattern use of lexicons	Lexicons	Number of terms	Example lexicon	Example terms
All rules	11	345	Body_parts	arm, eye, hair, teeth, toe, tongue, finger, fingers, nose
Group A1	7	105	A1_interact	interact, interactions, communicate, relationship
Group A2	3	72	A2_positive	severe, significant, pervasive, marked
Group A3	2	72	A3_object	door, toys, vacuum, blocks, book, television, lights
A1a	4	42	A1a_nonVerbalBehavior	eye contact, eye-to-eye gaze, gestures, nonverbal cues
A1b	2	11	A1b_consistent	good, consistent, appropriately, satisfactory
A1c	5	61	A1c_affect	excitement, feelings, satisfaction, concerns
A1d	12	159	A1d_engage	recognize, recognizes, reacts, respond, regard, attend
A2a	4	117	A2a_gained	gained, used, had, obtained, said, spoke
A2b	8	240	A2b_recepLang	direction, instructions, questions, conversations
A2c	7	145	A2c_idiosyncratic	breathy, echolalia, jargon, neologism, reduced
A2d	7	83	A2d_actions	actions, routines, play, signs, gestures, movements
A3a	7	106	A3a_obsess	obsessed, obsessive, perseverates, preoccupation
A3b	7	119	A3b_nonFunctionalPlay	stack, stacks, lines, lined, nonfunctional, arrange
A3c	3	67	A3c_abnormal	grind, grinds, rocks, twirls, spin, tap, clap, flap
A3d	3	43	A3d_sensitive	defensiveness, sensitivity, hypersensitivities
Total	92	1787	N/A^a^	N/A

^a^N/A: not applicable.

### Parser Development

We developed a rule-based parser to extract all A1, A2, and A3 rules as listed in the DSM. Each DSM group contains 4 specific rules that are representative of the criterion (A1a-d, A2a-d, and A3a-d). Our tool comprises 2 components: (1) annotation of relevant ASD trigger words in free text and (2) recognition of diagnostic criteria based on a pattern of trigger words.

The parser was developed through collaboration between NLP experts and clinicians. Annotations from EHRs were translated into patterns by NLP experts. Then, extensions, abstractions, and generalizations were discussed and the patterns augmented and expanded. This iterative process was continued until changes in patterns provided little or no improvement but increased error rates. Several development rounds were completed, and the EHRs were taken from the 2002 to 2010 surveillance years, with 53% of records having an ASD case status. The ASD label itself is of little consequence because both development and testing are done at the sentence level (not the record level). For testing, new EHRs were used that were not seen in previous development rounds. EHRs were selected randomly from those available to us.

#### Lexicons

Identifying ASD diagnostic criteria in text requires recognizing important trigger words (ie, words describing typical behaviors of ASD). We capture these words, as well as synonyms and singular or plural variants, in lexicons. Approximately 90 lexicons with about 20 terms each were manually created. Table 2 provides an overview with the examples of lexicons and the terms they contain. We used a lexical lookup for each term found in the text and annotated it with the lexicon’s label. These labels form part of the patterns used to describe DSM criteria (see the following text). Multiple patterns are needed to capture the different free text expressions for each DSM criterion.

The lexicons are optimized for patterns for each DSM criterion, so the same terms may appear in multiple lexicons. However, a few lexicons are shared by all patterns and used for different DSM criteria. Currently, there are 11 lexicons shared by all patterns (eg, the lexicons containing body parts). In addition, the patterns for the A1, A2, and A3 criteria share, respectively, 7, 3, and 2 lexicons. For example, DSM rules A1a, A1b, A1c, and A1d all require identification of “impairment in social interaction,” and the relevant terms for this trigger are combined in the lexicon “A1_interact.” In addition to these shared patterns, each DSM pattern requires additional individual lexicons optimized for that pattern.

#### Pattern Extraction

We used the General Architecture for Text Engineering (GATE) [30,31], a Java-based developer environment, to process the free text from EHRs. We chose GATE because it includes several standard NLP tools as well as the availability of its Java Annotation Pattern Engine (JAPE) to efficiently annotate patterns over text. First, standard processing is applied to all text:

- Tokenizer: recognize individual tokens in the text.
- Sentence splitter: set boundaries on sentences so that parts of speech can be deduced for each word in a sentence.
- POS tagger [32]: assign POS to each word, eg, noun, verb, and adjective. We used the Stanford Tagger.

**Figure 1:**
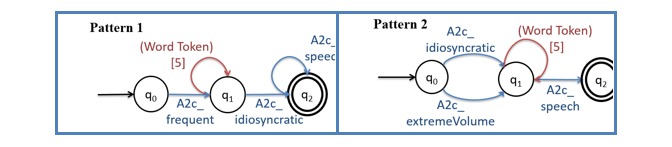
Visualization of 2 (of 7 existing) patterns for Diagnostic Manual of Mental Disorders criteria A2c.

After processing all free text, terms are annotated using gazetteer lookup. Using the term’s POS tags and lexical labels from the 92 lexicons, the annotated text is processed to identify matching patterns. POS tags help narrow down candidate terms, for example, “object” fits in our lexicons when it is a noun but not when it is a verb. Using 43 annotated records from the ADDSP containing 4732 sentences, we developed 12 sets of patterns (total 104 patterns) for the 12 DSM criteria (see Textbox 1).

Figure 1 shows two example finite state automata visualizing patterns for criterion A2c. Because we have many patterns of varying complexity, these examples are included to convey the general principle. A pattern starts in the q_0_ state, and when an appropriate input is presented, it proceeds to the next state. It is completed when a final state is reached. If no progress is possible, a sink state is reached and the process discarded. Each label on an arc (eg, A2c_speech) represents the lexicon of terms (terms indicating “speak” as relevant to rule A2c). For example, Pattern 1 would match the text “[often]_A2c_frequent_ [speaks]_word token_ [with]_word token_ [reduced]_A2c_idiosyncratic_ [volume]_A2c_speech._” The word “often” matches the lexicon “A2c_frequent,” along with words such as “frequently,” to indicate that this behavior is a regular occurrence. This match enables the transition from q_0_ to q_1_. The phrase “speaks with” is accepted in state q_1_, which accepts up to 5 word tokens that do not match other arcs transitioning out of this state. We empirically decided to allow 5 intervening terms to avoid the patterns becoming too specific while ensuring that elements in a pattern are still related in one underlying sentence. Then, “reduced” is accepted in the transition from q_1_ to q_2_ because it is included in “A2c_idiosyncratic” as one of the words to indicate abnormally low volume. (Lexicon “A2c_idiosyncratic” includes words that suggest atypical or idiosyncratic patterns of speech.) Finally, the word “volume” is accepted in q_2_ because it is one of the words related to speech that is included in lexicon “A2c_speech.”

All patterns are specified in a JAPE file. A JAPE file is a file where patterns to be annotated in the text can be described using GATE-specific formatting. GATE “reads” the JAPE files and applies them to text. When a pattern in the JAPE is recognized in the text, the text matching the pattern is annotated with the labels specified in the JAPE file.

## Results

### Parser Evaluation

#### Testbed

Our testbed consists of the 50 new EHRs, not used during development, containing 6634 sentences. The EHRs were randomly sampled from the 2000-2008 surveillance years, with 68% of records having positive ASD case status. Because evaluation is done at the sentence level and does not take record-level information into account, the case label itself is of little consequence. These are records that were annotated by the clinical experts and the text and annotation stored by us in electronic format. Of the entire set, 20.45% (1357/6634) sentences contained annotations, with some sentences containing more than 1 annotation.

A human-created gold standard, such as our testbed, is seldom completely perfect and consistent: entities may have been missed by the human annotators. We noticed such inconsistencies in prior work by us [33] and by others [21]. In this testbed, we encountered a few omissions, that is, annotations that were identified by our parser but not by the human annotators. This may be an oversight, or it may reflect the annotator’s intention not to annotate all phrases when they are almost identical and represent the same DSM criterion. While this may suffice for manual review, a complete gold standard is needed for automated evaluation of an algorithm. Therefore, we ensured that in our test phase, we could rely on a complete gold standard. In addition to the phrases annotated, we requested additional expert evaluation to verify whether patterns discovered by the parser but not annotated (false positives) should be part of the gold standard. Of the 366 plausible patterns identified by our system, 277 were identified by the experts as part of the gold standard. We added these missed annotations to our gold standard. Table 3 provides an overview of the number of annotations in the gold standard.

#### Metrics

Similar to evaluation standards by others [34], we accept partial matches, defined as machine annotations that are considered correct if they contain any part of a gold standard annotation. For example, in the sentence “He also exhibited poor eye contact with the examiner,” our tool annotated “exhibited poor eye contact,” while the human expert annotated “poor eye contact with the examiner.” We accepted these annotations because this region of text can identify meaningful information relevant to criterion A1a (nonverbal behaviors). These adjustments in our evaluation criteria reflect the high variety in expert annotations, which tend to be inconsistent in their inclusion of subjects and verbs within the boundaries of the annotations.

For our evaluation, we calculated 4 metrics. Precision provides an indication of how correct the annotations made by the parser are; in other words, if the parser annotates sentences with a DSM label, this refers to the percentage of the labels that are correct. Recall (also referred to as sensitivity) provides an indication of how many of the annotations the parser is able to capture; in other words, of all the sentences that received a DSM label by the human annotators, what percentage does the parser also label correctly. We also calculate the *F*-measure, which is the harmonic mean of recall and precision. The scores for the *F*-measure indicate how balanced an approach is: when recall and precision are similarly high, the *F*-measure will be high; however, if one of them is low, the *F*-measure will reflect this with a low F-score. Finally, we also calculate specificity, which indicates how well our parser can ignore sentences that are not an expression of DSM criteria.

Precision (or PPV) = (True Positive) ∕ (True Positive + False Positive)

Recall (or Sensitivity) = (True Positive) ∕ (True Positive + False Negatives)

*F*-measure = 2 × (Precision × Recall) ∕ (Precision + Recall)

Specificity = (True Negatives) ∕ (True Negatives + False Positive)

We calculate these metrics at the annotation and at the sentence level. A true positive at the *annotation level* means that the system identified a criterion-specific annotation within a sentence also present in the gold standard. If a record or sentence contains 2 annotations for the same criterion, both should be identified individually. This is a stringent evaluation. For example, the sentence “He makes minimal eye contact with adults and struggles with turn-taking in conversations” is evaluated separately for criteria A1a (minimal eye contact) and criteria A2b (turn-taking in conversations).

We also apply the *sentence-level* evaluation for information extraction. In this case, a true positive is defined as identifying the sentence that contains gold standard annotations for a criterion, and the system has identified at least 1 annotation for the same rule. This evaluation can be more forgiving when a sentence contains more than 1 annotation.

#### Results of Parser Evaluation

Table 4 shows the results. At the annotation level, we achieved 74% precision and 42% recall on average. We took the microaverage, which combines the true and false positive counts across all rules. For individual criteria, precision was higher (≥75%) for most except two (criterion A1d and A3d). Recall was also particularly low for these two criteria, along with A1b and A1c. The best precision and recall were achieved for criterion A1a, with more than half of the annotations (57% recall) identified and with very few errors (96% precision).

**Table 3:** Gold standard overview.

Diagnostic and Statistical Manual of Mental Disorders diagnostic criteria		Gold standard	
Rule	Theme	Total in records	Average per record
A1a	Nonverbal behaviors	126	2.52
A1b	Peer relationships	91	1.82
A1c	Seeking to share	37	0.74
A1d	Emotional reciprocity	165	3.3
A2a	Spoken language	406	8.12
A2b	Initiate or sustain conversation	333	6.66
A2c	Stereotyped or idiosyncratic language	127	2.54
A2d	Social imitative play	66	1.32
A3a	Restricted patterns of interest	62	1.24
A3b	Adherence to routines	135	2.7
A3c	Stereotyped motor mannerisms	68	1.36
A3d	Preoccupation with parts of objects	28	0.56
Total	N/A^a^	1644	32.88

^a^N/A: not applicable.

**Table 4:** Annotation-level results.

Annotations^a^	Total in gold standard (number of annotations^b^)	Evaluation		
		Precision	Recall	F-measure
A1a	126	0.96	0.57	0.72
A1b	91	0.63	0.27	0.38
A1c	37	0.78	0.19	0.30
A1d	165	0.62	0.27	0.37
A2a	406	0.69	0.44	0.53
A2b	333	0.79	0.44	0.57
A2c	127	0.68	0.36	0.47
A2d	66	0.79	0.56	0.65
A3a	62	0.83	0.40	0.54
A3b	135	0.75	0.51	0.61
A3c	68	0.82	0.41	0.55
A3d	28	0.53	0.29	0.37
Microaverage	N/A^c^	0.74	0.42	0.53

^a^Based on 6634 sentences.

^b^Total annotations=1644.

^c^N/A: not applicable.

**Table 5:** Sentence-level results.

Sentences^a^	Total in gold standard (number of sentences)^b^	Evaluation			
		Precision	Recall	F-measure	Specificity
A1a	120	0.97	0.59	0.74	1.00
A1b	90	0.68	0.30	0.42	1.00
A1c	35	0.78	0.20	0.32	1.00
A1d	158	0.63	0.28	0.39	1.00
A2a	391	0.71	0.45	0.55	0.99
A2b	329	0.83	0.47	0.60	1.00
A2c	121	0.67	0.37	0.48	1.00
A2d	65	0.83	0.58	0.68	1.00
A3a	61	0.73	0.36	0.48	1.00
A3b	123	0.74	0.52	0.61	1.00
A3c	64	0.82	0.42	0.56	1.00
A3d	28	0.53	0.29	0.37	1.00
Microaverage	1585	0.76	0.43	0.55	1.00
Any Rule	1357	0.82	0.46	0.59	0.97

^a^Based on 6634 sentences.

^b^Sentences with annotations =1357.

The results are very similar to those for the sentence-level evaluation (Table 5). Both metrics are slightly higher, with average precision at 76% and average recall at 43%. For the A1a criterion, more than half of the required sentences were identified (recall 59%) with minimal errors (97% precision). Using a sentence as a unit of analysis, it is also possible to compute specificity, or true negative rate, which was not possible with annotation-level evaluation because we would have to predefine in advance how many possible annotations (ie, sentence segments) there are in the EHRs. However, specificity is not a very interesting metric for this task. We achieve nearly perfect specificity because only 0.5%-5% of all sentences contain true annotations for each rule, and our system reports very few false positives (high precision).

We conducted a final, more lenient approach by evaluating whether the system can identify the relevant sentences for DSM criteria, regardless of which criterion they represent. In this case, we found that our parser achieves 82% precision and 46% recall in identifying the 1357 sentences that were annotated for autism-like behavior (Table 5, last line “Any rule”).

## Discussion

### Principal Findings

Overall, the rule-based approach resulted in a better performance than the machine learning approach. Some criteria, such as A1c and A3d, showed very large differences in precision between the two approaches, while others, like A1d and A3a, showed a large difference in recall. This may be due to the sparsity of the examples available for training. Furthermore, we chose to evaluate decision trees because of their interpretability. More sophisticated algorithms will be tested when larger datasets become available, and these may provide better results.

As is expected with the development of a rule-based extraction system, the results for precision are higher than those for recall. False negatives represent the annotations that were missed by our algorithm and lowered recall. We noticed 3 types of false negatives due to annotations not seen in the training data. First, there are new examples of behaviors; for example, “being a picky eater” is an A1a criterion, but it did not appear in our training data. To solve this, we will write additional JAPE rules. Second, there are sometimes different lexical variants of behaviors (ie, synonyms or related terms) used to describe behaviors. To solve this, we will look into expanding our lexicons, for example, through using word embeddings. Third, sometimes complex language or longer interstitial text is used that is not captured by our patterns. For example, “Eye contact, while it was also present, was limited at times.” The solution will require further augmenting the patterns. In addition, some false negatives are the results of localized patterns. The criteria annotated in the EHR are sometimes determined by the clinicians using information in the EHR context or the surrounding text. This is not covered by JAPE patterns because it does not appear in the same sentence or neighboring text.

False positives usually occur for either of two reasons: accidental matches to nonsensical sentence fragments or plausible phrases with insufficient context. For example, Pattern 1 in Figure 1 also incorrectly matches to “[frequently]_A2c_frequent_ [will]_word token_ [exhibit]_word token_ [difficulty]_word token_ [handling]_word token_ [loud]_A2c_idiosyncratic_,” a fragment in our training dataset (while not part of the DSM criteria, the phrase is commonly found in the records) that obviously cannot be a correct annotation. Meanwhile, the machine annotation “difficulty communicating with the teacher and peers” appears to indicate a failure to develop peer relationships as described in criterion A1b, but it is not accepted by domain experts because the criterion refers to challenges in social interactions, while this text fragment focuses on verbal communication.

We see large differences between the various DSM-IV criteria. For example, criterion A1c, which refers to “a lack of spontaneous seeking to share enjoyment, interests, or achievements with other people,” is expressed completely differently in the test set and was not captured by our rules. This is not surprising because A1c is the criterion for which we have the least amount of training data (averaging 0.5 annotations per record). Additionally, the criterion covers a wide range of behaviors that can be expressed in many different ways. The variations and lack of data make describing patterns very difficult. Criterion A1a, which is related to nonverbal communication, obtained relatively high precision and recall. This is because clinicians tend to describe nonverbal communication in unambiguous, self-contained phrases, such as “eye contact” and “nonverbal communication,” for which we can create precise patterns. For a similar reason, we also obtained good results for criteria A3a, A3c, and A3d, which are about abnormal interests, stereotypical actions, and tactile sensitivities, respectively. Some of the criteria (eg, A3c) have precision near 90%. Criteria A2a and A2b, which describe expressive and receptive language issues, are most prevalent among the rules. Combined, they account for >40% of the gold standard annotations. Taking advantage of the large sample of gold standard annotations, we were able to develop many and obtain relatively stable performance from development to testing.

In our case, we believe lower recall does not preclude useful applications of the parser. While some particular expression of a DSM criterion may be missed, it will be rare that all expressions of that particular DSM criterion in one record would be missed and, so, the detected DSM criterion would be taken into account for case assignment. Moreover, because of the high precision of the parser, when an expression of a DSM criterion is flagged, it is unlikely to be a false positive. As a result, large-scale analyses that focus on patterns of different criteria can be performed.

### Parser Application: Case Study

#### Testbed

Given the high precision of our parser, we conducted a case study that shows insights into and the potential of the parser for future work. Our goal is to provide a broad overview of DSM criteria patterns found in existing EHRs over a 10-year span.

**Figure 2:**
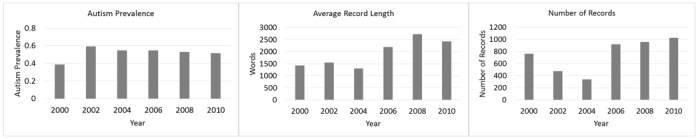
Descriptive information on 4480 records available electronically from the Arizona Developmental Disabilities Surveillance Program.

For our case study, we analyzed 4480 records available electronically from the ADDSP. These records have not been used during the development of the parser and contain a minimum of text (40 characters was empirically determined as the cutoff in this set; this represented about 10 words or a complete sentence, which is required for a complete annotation). We focus only on the free text fields and the results from applying our parser. Figure 2 shows the descriptive statistics. Records were collected every 2 years, starting in 2000 and ending (for our analysis) in 2010. In the first 3 collection periods, fewer records were collected; however, in each of the last 3 collection periods, around 1000 records were collected. The prevalence of autism in the records was lower in the first year (39%). This is associated with the relative inexperience of the data collection team who abstracted more records than necessary to avoid missing cases. In subsequent years, data collection was more efficiently focused on records that included information consistent with an autism diagnosis, and the proportion of children abstracted who were determined to have met the case definition was commonly between 50% and 60%.

Abstractor training has been consistent over the years with the goal to enter only the necessary information to meet the project deadlines. Even so, the average length of the free text has increased over the years: the average number of words before 2006 was 1427 and has increased to 2450 from 2006 until 2010, nearly double the number.

#### Results

**Figure 3:**
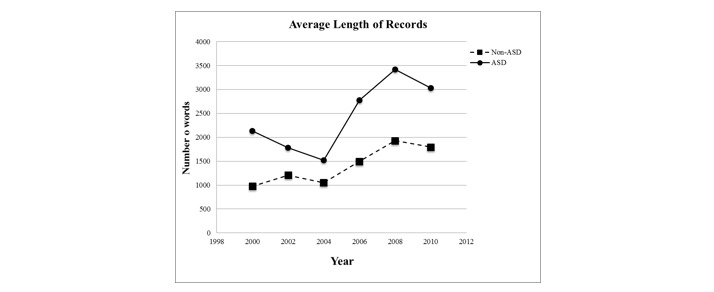
Electronic health record word count for autism spectrum disorder (ASD) and non-ASD cases.

The records contained on average 5.76 different DSM criteria. We performed our analysis separately for records of children with ASD and of those labeled as non-ASD. All counts are normalized by record length: the number of criteria found is divided by the number of words in the document. This normalization avoids increasing the count of criteria solely due to having longer records, for example, when a child is seen multiple times for evaluation and the resulting EHR is longer, but the diversity of criteria may remain the same. Figure 3 shows the word count for the EHRs used in this evaluation. The word counts for ASD and non-ASD cases follow a similar trend. After 2004, there has been an increase in the length of records, which levels off the next year.

**Figure 4:**
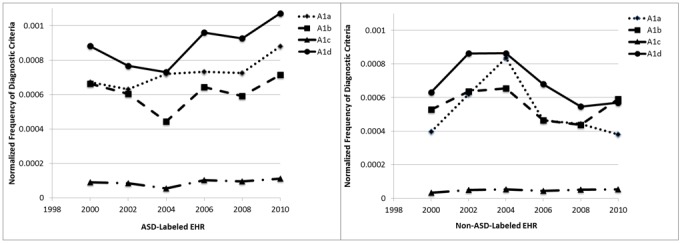
Average A1 criteria per record. ASD: autism spectrum disorder; EHR: electronic health record.

We first focus on the A1 DSM criteria. These criteria describe impairments in social interaction. For children with ASD (Figure 4, left graph), the A1d criterion (social or emotional reciprocity) is the most common criterion found in the records. The least commonly found criterion was the A1c criterion (shared interest). In the last 4 years, the average number of A1a, A1b, and A1d criteria described in the records has increased, but no similar increase in the average number of records containing A1c was observed.

We performed the same analysis for children without ASD (Figure 4, right graph). The results show, as expected, that fewer criteria are recorded in their records; the patterns are also different. The number of criteria recorded shows a decreasing trend over the last 4 years of records.

**Figure 5:**
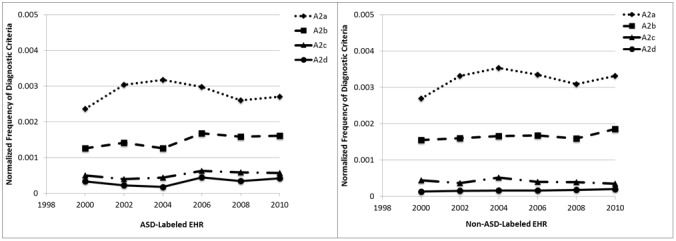
Average A2 criteria per record. ASD: autism spectrum disorder; EHR: electronic health record.

We repeat the same analysis for A2 DSM criteria (Figure 5), which focus on impairments in communication. The changes for A2 criteria are very small over the years. The most commonly found criterion is A2a (spoken language), and the least commonly found criteria are A2c (stereotyped or repetitive or idiosyncratic language) and A2d (imaginative play). There is a slight increase in 2002 and 2004 for the records of children with ASD, but few changes over the collection years. The total number of these criteria is higher than that of A1 criteria (see y-axis). Interestingly, there is little difference between the number of criteria found in EHRs labeled as ASD versus non-ASD.

**Figure 6:**
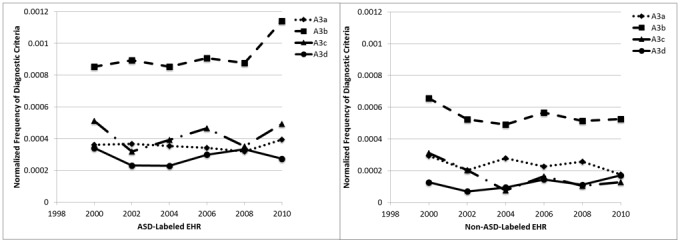
Average A3 criteria per record. ASD: autism spectrum disorder; EHR: electronic health record.

Finally, we show the analysis for A3 DSM criteria (Figure 6), which focus on restricted, repetitive, and stereotyped behavior patterns. For the records labeled as ASD, the most commonly found criterion is A3b (adherence to routines), with the other three criteria being less common and comparable to each other. Overall, fewer criteria are found in the non-ASD-labeled records.

#### Discussion

The presence of a criterion in a record depends first on its presence in the child, second on whether the evaluator notes that criterion in the child, and third on whether the evaluator notes it in the record. The criteria that we identified with the greatest frequency were A2a (spoken language) and A2b (initiate or sustain conversation). Issues with language acquisition are the most frequently noted first cause of parental concern [35-39]. It is standard to make a note of the reason for the evaluation, and this would be expected to typically capture the first parental concern. Furthermore, these A2 criteria are frequent in all records because most children evaluated for ASD exhibit some type of impairment in communication. Criteria that we found least frequently include A2c (stereotyped or repetitive or idiosyncratic language) and A2d (imaginative play). While these are classic characteristics of ASD, they are less well noted by parents.

The frequencies of criteria in the ASD case records were not as different from the non-ASD records as may be expected. However, all children whose records were included in data collection had some type of diagnosis or special education qualification; no typically developing children are included in these data [40]. Individual criteria for ASD may be seen in children with other developmental disabilities, but it is the constellation of criteria that defines ASD.

Some changes across the years of data collection were observed. The first was an increase in the number of words per record. This increase is likely to reflect a true increase in the words rather than any changes in data collection procedures, as increasing numbers of records to review have motivated efforts to improve efficiency and eliminate the collection of superfluous text. An increase in the number of criteria included will necessarily mean that more words are collected. Next was an increase in the frequency of some specific criteria among the ASD cases. Changes through time in the frequency of a specific criterion may reflect more children who exhibit the criterion or that evaluators may have a heightened awareness of the criterion and are, therefore, more likely to note it. Criteria that were increasing in frequency included A1a (nonverbal behaviors), A1d (social or emotional reciprocity), and A3b (adherence to routine), but the increase in A3b was noted only in the most recent year.

The increases in the frequencies of some criteria in this dataset contrast with results from a study in Sweden, which found fewer autism symptoms among children diagnosed in 2014 than among those diagnosed in 2004 [41]. Arvidsson et al have suggested that clinical diagnoses of autism are being made in the year 2014 for cases that are less severe and would not have been given that diagnosis in 2004. They further suggested that this may explain some of the increase in the estimated prevalence of ASD. Increases in the estimated prevalence in the ADDSP dataset, from 6.5 per 1000 in 2000 to 15.7 per 1000 in 2010, are not susceptible to this decrease in severity as our criteria for determining case status has been consistent over the time period. In fact, we observe an increased proportion of cases with certain criteria and an increase in the average number of criteria over time. The increased prevalence that we have estimated would reflect a decrease in the severity of the condition only if evaluators in the recent years are making a notation of symptoms that are so mild they would not have noted them earlier.

The trend of increasing frequency of criteria A1a (nonverbal behaviors) and A1d (social or emotional reciprocity) in ASD-labeled records and the decreasing trend in those same criteria in non-ASD-labeled records may represent improvements in evaluators’ awareness of these as symptoms of ASD and the importance of documenting these criteria for children who have the characteristics of ASD cases.

### Conclusion

We described the design and development of a rule-based NLP tool that can identify DSM criteria in text. In comparison to a baseline machine learning approach that used decision trees, the rule-based approach performed better. We evaluated our approach at the annotation level (ie, matching to each rule within a sentence) and at the sentence level (ie, matching to the correct sentence). The system performed reasonably well in identifying individual DSM rule matches, with approximately half of all individual criteria-specific annotations discovered (44% recall) with few errors (79% precision). As expected with manually developed rules, precision was high, while recall was lower. In future work, we intend to increase both lexicons and patterns using machine learning approaches while retaining human-interpretable rules. This will increase the recall of our system. Furthermore, we intend to add negation as an explicit feature, which we believe will be necessary to maintain high precision.

We demonstrated our parser on almost 5000 records and compared the presence of different DSM criteria across several years. Changes in document length as well as in the presence of different DSM criteria are clear. Our analysis also showed that some DSM criteria are almost equally present in both ASD and non-ASD cases. In the future, we intend to increase the size of our records and combine the information extracted (ie, the DSM criteria matches) with other data from the structured fields in those EHRs as well as combine the information with external databases containing environmental and other types of data.

Our future work will be 2-fold. First, we will investigate the integration of our system into the surveillance workflow. For maximum usefulness, we will aim at extreme precision or extreme recall (while both are desirable, there tends to be a trade-off). With extremely high precision, the extracted diagnostic criteria can be used to make case decisions with high precision. Labeling a case as ASD can be automated for a large set of EHRs; only the set where no ASD label is assigned would require human review (due to low recall). In contrast, with extremely high recall, cases where diagnostic criteria are not extracted can be labeled as non-ASD with high confidence and only the cases where a label of ASD is assigned would need review (due to low precision). Second, because the development time of a rule-based system is substantial and application to a new domain would require starting over, we will investigate leveraging lessons learned from the parser to a machine learning approach that can transfer to different domains in mental health.

## Abbreviations

ADDSP: Arizona Developmental Disabilities Surveillance Program
ASD: autism spectrum disorder
CDC: Centers for Disease Control and Prevention
DSM-IV-TR: Diagnostic Statistical Manual of Mental Disorders, Fourth Edition, Text Revision
EHR: electronic health record
GATE: General Architecture for Text Engineering
ICD: International Classification of Diseases
JAPE: Java Annotation Pattern Engine
NLP: natural language processing
POS: part-of-speech
UMLS: Unified Medical Language System
